# Qualitative and semi-quantitative evaluation of myocardium perfusion with 3 T stress cardiac MRI

**DOI:** 10.1186/s12872-015-0159-1

**Published:** 2015-12-07

**Authors:** Chun-Ho Yun, Jui-Peng Tsai, Cheng-Ting Tsai, Greta S. P. Mok, Jing-Yi Sun, Chung-Lieh Hung, Tung-Hsin Wu, Wu-Ta Huang, Fei-Shih Yang, Jason Jeun-Shenn Lee, Ricardo C. Cury, Anas Fares, Lemba Dina Nshisso, Hiram G. Bezerra

**Affiliations:** Department of Biomedical Imaging and Radiological Sciences, National Yang Ming University, 155 Li-Nong St., Sec. 2, Taipei, 112 Taiwan; Department of Radiology, Mackay Memorial Hospital, No. 92, Sec 2, Chungshan N. Rd, Taipei, 104 Taiwan; Division of Cardiology, Department of Internal Medicine, Mackay Memorial Hospital, Taipei, Taiwan; Biomedical Imaging Laboratory, Department of Electrical and Computer Engineering, Faculty of Science and Technology, University of Macau, Macau, SAR China; Cardiovascular MRI and CT Program, Baptist Cardiac Vascular Institute, Miami, FL USA; Cardiovascular Department, University Hospitals Case Medical Center, Cleveland, USA

**Keywords:** Quantitative, Qualitative, Myocardial perfusion, 3-T, MRI

## Abstract

**Background:**

3 T MRI has been adopted by some centers as the primary choice for assessment of myocardial perfusion over conventional 1.5 T MRI. However, there is no data published on the potential additional value of incorporating semi-quantitative data from 3 T MRI. This study sought to determine the performance of qualitative 3 T stress magnetic resonance myocardial perfusion imaging (3 T-MRMPI) and the potential incremental benefit of using a semi-quantitative perfusion technique in patients with suspected coronary artery disease (CAD).

**Methods:**

Fifty eight patients (41 men; mean age: 59 years) referred for elective diagnostic angiography underwent stress 3 T MRMPI with a 32-channel cardiac receiver coil. The MR protocol included gadolinium-enhanced stress first-pass perfusion (0.56 mg/kg, dipyridamole), rest perfusion, and delayed enhancement (DE). Visual analysis was performed in two steps. Ischemia was defined as a territory with perfusion defect at stress study but no DE or a territory with DE but additional peri-infarcted perfusion defect at stress study. Semi-quantitative analysis was calculated by using the upslope of the signal intensity-time curve during the first pass of contrast medium during dipyridamole stress and at rest. ROC analysis was used to determine the MPRI threshold that maximized sensitivity. Quantitative coronary angiography served as the reference standard with significant stenosis defined as >70 % diameter stenosis. Diagnostic performance was determined on a per-patient and per-vessel basis.

**Results:**

Qualitative assessment had an overall sensitivity and specificity for detecting significant stenoses of 77 % and 80 %, respectively. By adding MPRI analysis, in cases with negative qualitative assessment, the overall sensitivity increased to 83 %. The impact of MPRI differed depending on the territory; with the sensitivity for detection of left circumflex (LCx) stenosis improving the most after semi-quantification analysis, (66 % versus 83 %).

**Conclusions:**

Pure qualitative assessment of 3 T MRI had acceptable performance in detecting severe CAD. There is no overall benefit of incorporating semi-quantitative data; however a higher sensitivity can be obtained by adding MPRI, especially in the detection of LCx lesions.

## Background

Magnetic resonance myocardial perfusion imaging (MRMPI) is a well-established technique for noninvasive detection of myocardial ischemia due to coronary stenosis. Compared with single-photon emission computed tomography (SPECT), the most widely used technique, MRMPI has superior spatial resolution that could facilitate differentiation of subendocardial and transmural perfusion defects. Additionally, MRMPI has fewer artifacts and is free from ionizing radiation. The vast majority of data available on MRMPI has been obtained using a 1.5-T scanner. However, with the newly available 32-channel cardiac coil and new acquisition strategies such as k-space and time sensitivity encoding (k-t SENSE), 3-T MRI system provides increased signal-to-noise ratio (SNR), reduced imaging time, and an even greater spatial resolution.

**Fig. 1:**
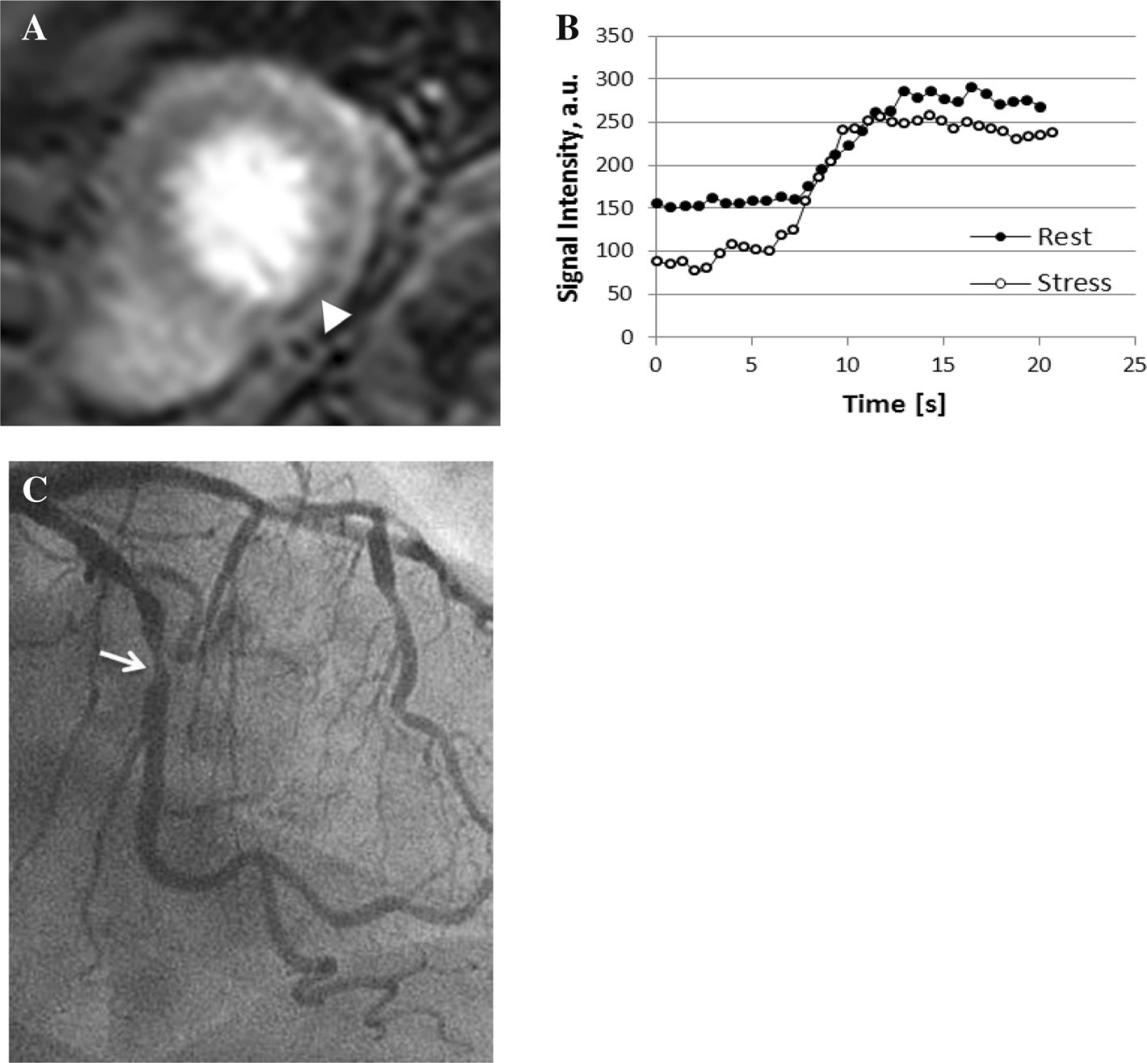
**a** short axis view of stress MR perfusion showing perfusion defect in the inferior wall segment (arrowhead). **b** corresponding rest and stress semi-quantitative evaluation. The inferior segment shows decreased signal intensity during stress **c** coronary angiography of the patient showing a stenosis of 74 % in the mid LCx (arrow)

Over the past decade, qualitative analysis has been the most commonly used method for MRMPI in the clinical practice. Although studies have demonstrated that both qualitative and semi-quantitative assessments of myocardial perfusion, using a 1.5-T scanner, have moderate diagnostic accuracy for the detection of coronary artery disease (CAD) [1–3], quantitative MRMPI has the added advantage of operator-independence and has yielded a decrease in inter- and intra-observer variability.

The aim of this study is to investigate the performance of 3-T stress MRMPI and the potential additional value of semi-quantification of myocardial flow for the detection of significant CAD.

## Methods

### Study population

The study was approved by the Institutional Review Board of Mackay Memorial Hospital, Taipei, Taiwan. Sixty-four patients were recruited between September 2009 and December 2010. All patients were referred for invasive coronary angiography due to clinically suspected or known CAD. Reasons for exclusion included high-degree atrioventricular block, prior coronary artery bypass surgery, pregnancy, hypotension (systolic blood pressure < 90 mmHg), decompensated congestive heart failure (New York Heart Association functional class III or IV), and standard contraindications to MRI imaging, dipyridamole, or gadolinium. All patients were advised not to consume caffeine within 24 h of the study. The written informed consent was obtained from all enrolled patients.

### MRI protocol

All studies were performed on a 3-T MRI (Achieva 3 T, Philips Medical Systems, Best, The Netherlands) equipped with 80 mT/s maximum field gradients and a 200 T/m/s slew rate using a 32-channel cardiac receiver coil (Invivo, Gainesville, FL) and ECG synchronization. For myocardial perfusion imaging, a saturation recovery gradient-echo T1 weighted sequence (T1-TFE) was used with prospective ECG triggering; sequence parameter included: non-selected shared 90 saturation pulse, TR/TE/flip angle: 2.2 ms/0.8 ms/10°, spatial resolution 1.8x1.8x8 mm, matrix 156x192, Sense factor 2.5–3. After the scout images for cardiac geometry and acquisition of three standard short axis views (apical, mid and basal) of left ventricle, the dipyridamole infusion (0.56 mg/kg; total duration 4 min) was started with an infusion pump (Medrad, Indianola, PA). The stress MRMPI was performed six minutes after the beginning of dipyridamole infusion with 0.05 mmol/kg gadolinium (Multihance, Bracco) injected intravenously with an injection rate of 4 ml/s, followed by a 25 ml saline flush at the same rate. Images were acquired for 60 cardiac phases. After stress MRMPI, aminophylline was given (3 mg/kg) intravenously and delivered over 2 min. Ten minutes later, rest MRMPI was performed using the identical protocol. Then the following 0.05 mmol/kg gadolinium was injected to achieve total dose of 0.15 mmol/kg. Approximately 10 min later, delayed-enhanced MR imaging (DE-MR) was performed by using the inversion-recovery prepared gated fast gradient-echo sequence (1.5x1.7x5mm).

### Visual MRI analysis

Two experienced readers, blinded to all data including clinical information and angiographic results, visually analyzed all MRI images. In cases of disagreement, consensus was achieved by use of a third reader.

Perfusion analysis of each myocardial segment (except the apex) was performed based on the 17-segment model recommended by the AHA (American Heart Association) [4]. Ischemia was defined as a territory with perfusion defect at stress MRMPI but no DE-MR or a territory with DE-MR but additional peri-infarcted perfusion defect at stress MRMPI. Diagnostic performance was determined on a per-patient and per-vessel basis.

### MPRI (Semi-quantitative MRI) analysis

All MRI images were sent to the Harrington Heart and Vascular Institute Cardiovascular Imaging Core Laboratory (Cleveland, Ohio) for semi-quantitative perfusion analysis. Analyses were performed using dedicated software (CAAS-MRV, version 3.2.1, Pie Medical, Maastricht, The Netherlands) by examiners who were blinded to angiographic results. Perfusion analysis was performed to obtain signal intensity for each myocardial segment over time. Endocardial and epicardial contours were drawn semi-automatically and propagated through all slices on all images, then manually adjusted for breathing. Mean signal intensity was registered over time. A Signal intensity-time curve was generated from transmural myocardium for all segments on consecutive images before and during the contrast medium administration. The LV input function was determined by obtaining the signal intensity-time curve of basal level left ventricular cavity. In each signal intensity-time curve, the maximal initial slope was determined by the software.

LV myocardium was divided into 6 segments per slice, and then further divided into 16 AHA segments. Myocardial perfusion reserve index (MPRI) was calculated by dividing the results of segmental upslope at stress through rest. All segments were assigned to the respective perfusion territory. The mean of the two lowest scoring segments was assigned to each perfusion territory for further analysis.

### Analysis of adding semi-quantitative on qualitative results

In order to exam the incremental value of semi-quantitative analysis for clinical application, we selected the segments with negative results by qualitative assessment and re-stratified them according to the semi-quantitative results.

### Coronary angiography and Quantitative Coronary Analysis (QCA)

Less than two months after the MRI examination, all patients underwent conventional coronary angiography by radial approach. Multiple cine angiographic projections were recorded on a hard drive and subsequently sent to the Harrington Heart and Vascular Institute Cardiovascular Imaging Core Laboratory for quantitative coronary analysis (QCA). An expert who was blinded to visual and quantitative CMR performed analysis using dedicated offline software (CAAS II Analysis System; Pie Medical, Maastricht, the Netherlands). Quantitative coronary angiography was used as the gold standard and was performed on all vessels to characterize stenosis severity. The percentage diameter of stenosis was calculated using an automated contour detection algorithm with at least two orthogonal angiographic views. Vessels with at least one coronary lesion of >70 % diameter stenosis were considered significant. For further analysis, the coronary tree was divided into 16 segments for comparison with CMR. Territory dominance was taken into consideration (Fig. 1).

### Statistical analysis

Descriptive statistics and comparisons were obtained using SAS 9.2 (SAS Institute Inc., Cary, NC, USA.). Continuous variables were expressed as mean ± SD. Categorical variables are expressed as numbers and percentages. Non-normally distributed variables were compared using the Wilcoxon two-sample test. Normally distributed variables were compared using the Student’s *t*-test. In order to take into account multiple measurements per subject, vessel level analysis was performed using generalized estimating equations. Kappa values were calculated to compare inter-observer agreement for perfusion defects of myocardial territories on a per-patient and per-vessel basis.

Receiver operating characteristics (ROC) analyses were performed using R version 2.13.1 (The R Foundation for Statistical Computing (C) 2011). ROC was used to assess the diagnostic accuracy of visual MRI and determine the MPRI threshold that maximized sensitivity and specificity. Diagnostic performance was determined on a per-patient and per-vessel basis. The Incremental improvement of MPRI on visual MRI is presented as sensitivity, specificity, positive predictive value and negative predictive value.

## Results

### Incremental value of MPRI

MRI was successfully performed without any side effects related to gadolinium or dipyridamole in 64 patients. A total of 58 patients (Table 1) had CMR results available for MPRI calculation, as 6 patients were excluded due to poor image quality, due to the presence of significant artifact. A total of 162 coronary territories were available for analysis. There were a total of fifty coronary territories with perfusion defects and twelve with delayed enhancement (DE). In forty-three coronary territories with perfusion defect but no DE, thirty two territories showed subendocardial perfusion defects (involving less than 50 % of LV wall thickness) and the rest showed transmural perfusion defects.

**Table 1:** Patient clinical characteristics

	*N* = 58
Demographics	
Gender: (Female/Male)	17/41
Age (yrs)	59.47 ± 10.66
Body Weight (kg)	69.63 ± 12.56
Height (cm)	161.54 ± 10.49
BMI (kg/m2)	26.78 ± 5.34
Medical history, n (%)	
Smoker	16/58 (28 %)
Former smoker	13/58 (22 %)
Diabetes	15/58 (26 %)
Hypertension	34/58 (59 %)
Dyslipidemia	28/58 (48 %)
Family History of CAD	24/58 (41 %)
Cerebral vascular accident	2/58 (3 %)
Known history of CAD	33/58 (57 %)
Known history of Angina	22/58 (38 %)
Known history of myocardial infarction	9/58 (16 %)
Know history of PCI or stent implantation	6/58 (10 %)
Lab data	
Total Cholesterol, mg/dl	186.17 ± 40.23
Triglyceride, mg/dl	141.09 ± 70.40
LDL, mg/dl	113.36 ± 32.55
HDL, mg/dl	41.57 ± 19.34
Medication, n (%)	
Aspirin	35/58 (60 %)
ß-blocker	27/58 (47 %)
Statin	30/58 (52 %)
CAD classification	
One vessel	10/58 (17 %)
Two vessel	7/58 (12 %)
Three vessel	1/58 (2 %)
None	40/58 (69 %)
Hemodynamic data	
Heart rate at stress (beats/min)	78.21 ± 8.55
Heart rate at rest (beats/min)	71 ± 8.07
Systolic BP at stress (mmHg)	126.81 ± 14.82
Diastolic BP at stress (mmHg)	79.67 ± 9.85

*LDL* Low-density lipoprotein, *HDL* High-density lipoprotein, *PCI* percutaneous coronary intervention

The prevalence of significant coronary artery stenosis (>70 %) was 31 % (18 of 58 patients), including ten patients with single-vessel disease, seven patients with double-vessel disease and one patient with triple-vessel disease. At the patient level, using QCA as the gold standard, qualitative MRI had a sensitivity and specificity of 77  and 80 % respectively. ROC analyses resulted in a MPRI value of 0.818, providing the optimal sensitivity and specificity to detect CAD at >70 % stenosis of 58 and 74 %, respectively, and a positive and negative predictive value of 52  and 78 % (AUC of 0.63), respectively. The additional information provided by the semi-quantitative MRI data produced some improvement in the sensitivity but a disproportional loss of specificity. At a patient level analysis, the use of MPRI resulted in an increase in sensitivity (83 vs. 77 %), but a decrease in specificity (63 vs. 80 %). Similarly, at the vessel level, the use of MPRI information produced a small increase in sensitivity (84 % vs. 76 %) but a larger loss of specificity (77 % vs.91 %) (Table 2). Further stratification by territory revealed the same pattern of gain in sensitivity associated with a loss in specificity. For example, a sub-analysis of diagnostic performance in the LCx territory showed an increase in sensitivity from 66 to 83 %, with a decrement in specificity from 93 to 75 % (Table 3).

**Table 2:** Sensitivity and specificity of qualitative MRI with and without additional MPRI information

	Patient level		All vessels	
	Qualitative	MPRI	Qualitative	MPRI
Sensitivity	77 %	83 %	76 %	84 %
Specificity	80 %	63 %	91 %	77 %

*MPRI* myocardial perfusion reserve index

**Table 3:** Sensitivity and specificity of qualitative MRI with and without additional MPRI information by vessel level

	LAD		LCx		RCA	
	Qualitative	MPRI	Qualitative	MPRI	Qualitative	MPRI
Sensitivity	80 %	90 %	66 %	83 %	77 %	77 %
Specificity	92 %	79 %	93 %	75 %	88 %	81 %

MPRI cutoff: 0.818

*LAD* left anterior descending, *LCx* left circumflex, *RCA* right coronary artery, *MPRI* myocardial perfusion reserve index

### Inter-observer agreement

Of the 58 patients, there was agreement in the determination of myocardial ischemia in 52 (90 %,κ = 0.79) on a per-patient basis. For coronary territories, there was agreement in 52 patients for LAD (90 %,κ = 0.72), 53 patients for LCx (91 %,κ = 0.69) and 53 patients for RCA (91 %,κ = 0.77).

## Discussion

This study is the first to report on the diagnostic performance of 3-T MRMPI combining visual and semi-quantitative analysis in detecting significant coronary stenosis in symptomatic patients. The main findings of our study are as follows: First, qualitative 3-T MRMPI has moderate accuracy in detecting significant coronary artery stenosis defined as >70 % luminal narrowing. Second, semi-quantitative assessment of 3-T MRMPI is specific but with low sensitivity. Third, the addition of semi-quantitative methods in cases with initial negative results on visual assessment provided superior sensitivity but, with decreased specificity. Forth, the impact of adding semi-quantitative on qualitative assessment was most effective for the LCx territory. Most importantly, we demonstrated that a stepwise strategy of applying semi-quantification in an initially qualitatively negative assessment could improve the sensitivity for detection of significant CAD, highlighting the potential utility as a screening tool.

SPECT myocardial perfusion imaging plays a major role in the field of cardiovascular imaging for detecting physiologically significant CAD. It also provides robust prognostic information to physicians and patients [5–7]. However, SPECT has several limitations. There is poor spatial resolution that can lead to false negative results in the setting of balanced ischemia due to multi-vessel disease. Also, attenuation artifacts are a common occurrence in obese patients and women [8]. Prior studies of 1.5-T MRMPI have shown that it yields high diagnostic accuracy compared to quantitative coronary angiography in detection of coronary artery disease while avoiding ionizing radiation exposure [9]. However, 3-T MRI is a relatively unpopular technique in cardiac imaging due to inhomogeneity in the high magnetic field, and increased susceptibility of artifacts [10]. With double the magnetization from 1.5-T to 3-T, a higher signal-to-noise ratio, and better tissue contrast [11], 3-T MRMPI shows promise for a future role in clinical practice. Recently, 3-T MR myocardial perfusion was compared head to head with 1.5-T, and 3 T MR and showed superior diagnostic accuracy in prediction of single and multivessel disease with increased contrast-to-noise and tissue-to-noise ratios [12].

Clinically, qualitative analysis is the most common method used in MR myocardial perfusion. There are only few studies [12–14] directly comparing pure qualitative 3-T MR myocardial perfusion with significant coronary stenosis (>70 %) on angiography. One study reported sensitivity of 89 to 100 %, specificity of 55 to 79 % and diagnostic accuracy of 77 to 85 % in per-patient level analyses as compared with 77 % sensitivity, 80 % specificity and 79.3 % accuracy in the current study [12, 14, 15]. However, qualitative assessment of MRMPI in 1.5-T has been reported to be operator-dependent with inter-observer variability as well as inferior performance compared to semi-quantitative assessment [16, 17]. Our results revealed the improvement of sensitivity by combing qualitative and semi-quantitative analysis in 3-T MRMPI.

Nevertheless, the observed increase in sensitivity by adding the semi-quantitative assessment on the initial negative results cases in visual assessment cannot translate into an improved diagnostic accuracy due to a significant decrease in specificity. This might be explained by several factors. First, semi-quantitative analysis is reported superior to visual analysis in diagnostic performance but still is a time consuming and cumbersome post-processing method, and it has rarely been performed in clinical practice. Therefore, we only applied semi-quantitative assessment on initial negative results cases from visual assessment. Secondly, previous studies have demonstrated that the diagnostic performance of stress MR myocardial perfusion depends on the severity of coronary stenosis detected on QCA [12, 18]. Cheng et al. [12] showed a reduced specificity for detection of stenosis of ≥70 % versus stenosis of ≥50 % in 3.0 T perfusion CMR. Klein et al. [18] also found that the detection of coronary stenosis of ≥70 %, as opposed to ≥50 %, resulted in a reduction in specificity from 63 % to 58 % in a 1.5 T study combining both perfusion and LGE imaging in a visual interpretation. By comparison, Ikuye et al. [19] showed good to moderate value in diagnostic accuracy in LAD and RCA but poor value in LCx territory by semi-quantitative assessment of 3-T myocardial perfusion, compared with quantitative coronary angiography ≥70 % stenosis. Our results demonstrated the most significant improvement in sensitivity but with moderate to severe decrement in specificity in the LCx territory, in sub-analysis at the vessel level, when combining qualitative and semi-quantitative analysis.

### Limitations

There were several limitations of the present study. First, coronary angiography is not a truly gold reference standard for myocardial perfusion images but remains the most established technique for decision making in interventional cardiology. In coronary lesions with stenosis over 70 % but, with normal myocardial perfusion, the diagnostic performance might not be assessed accurately. On the other hand, the stenosis grade of angiography cannot provide a reference in the setting of microvascular disease leading to CMR perfusion defect. Second, we did not compare CMR perfusion with SPECT, the most popular myocardial perfusion technique or other advanced functional studies including invasive fractional flow reserve (FFR), stress PET, stress/FFR CT. Third, the dose of dypiridamole in our study is not a high dose protocol which may fail to induce myocardial perfusion defects in some cases. Finally, we did not perform full-quantification of MR myocardial perfusion, which demonstrated close correlation to the results of FFR in hemodynamic significance of coronary artery disease.

## Conclusion

The results of our study show that performance of the pure qualitative assessment 3-T cardiac MRI in detecting significant coronary artery disease is acceptable. By adding semi-quantitative analysis, the sensitivity was increased, particularly in the detection of LCx lesions.

## Abbreviations

CAD: Coronary artery disease
CMR: Cardiac magnetic resonance imaging
CT: Computed tomography
DE-MR: Delayed-enhanced MR imaging
ECG: Electrocardiogram
FFR: Fractional flow reserve
LAD: Left anterior descending artery
LCx: Left circumflex artery
LV: Left ventricle
MRMPI: Magnetic resonance myocardial perfusion imaging
MPRI: Myocardial perfusion reserve index
RCA: Quantitative coronary analysis
RCA: Right coronary artery
PET: Positron emission tomography
SPECT: Single-photon emission computed tomography

## References

[1] Schwitter J, Nanz D, Kneifel S, Bertschinger K, Buchi M, Knusel PR (2001). Assessment of myocardial perfusion in coronary artery disease by magnetic resonance: a comparison with positron emission tomography and coronary angiography. Circulation.

[2] Nagel E, Klein C, Paetsch I, Hettwer S, Schnackenburg B, Wegscheider K (2003). Magnetic resonance perfusion measurements for the noninvasive detection of coronary artery disease. Circulation.

[3] Arai AE, Gaither CC, Epstein FH, Balaban RS, Wolff SD (1999). Myocardial velocity gradient imaging by phase contrast MRI with application to regional function in myocardial ischemia. Magn Reson Med.

[4] Cerqueira MD, Weissman NJ, Dilsizian V, Jacobs AK, Kaul S, Laskey WK (2002). Standardized myocardial segmentation and nomenclature for tomographic imaging of the heart. A statement for healthcare professionals from the Cardiac Imaging Committee of the Council on Clinical Cardiology of the American Heart Association. Circulation.

[5] Smith SC, Feldman TE, Hirshfeld JW, Jacobs AK, Kern MJ, King SB (2006). ACC/AHA/SCAI 2005 guideline update for percutaneous coronary intervention: a report of the American College of Cardiology/American Heart Association Task Force on Practice Guidelines (ACC/AHA/SCAI Writing Committee to Update 2001 Guidelines for Percutaneous Coronary Intervention). Circulation.

[6] Patel MR, Dehmer GJ, Hirshfeld JW, Smith PK, Spertus JA, American College of Cardiology Foundation Appropriateness Criteria Task F (2009). ACCF/SCAI/STS/AATS/AHA/ASNC 2009 Appropriateness Criteria for Coronary Revascularization: a report by the American College of Cardiology Foundation Appropriateness Criteria Task Force, Society for Cardiovascular Angiography and Interventions, Society of Thoracic Surgeons, American Association for Thoracic Surgery, American Heart Association, and the American Society of Nuclear Cardiology Endorsed by the American Society of Echocardiography, the Heart Failure Society of America, and the Society of Cardiovascular Computed Tomography. J Am Coll Cardiol.

[7] Windecker S, Kolh P, Alfonso F, Collet JP, Cremer J, Falk V (2014). Task Force on Myocardial Revascularization of the European Society of Cardialogy, the European Association for Cardio-Thoracic Surgery Developed with the special contribution of the European Association for Percutaneous Cardiovascular Interventions. Eur Heart J.

[8] Schwaiger M (1994). Myocardial perfusion imaging with PET. J Nucl Med.

[9] Hamon M, Fau G, Nee G, Ehtisham J, Morello R, Hamon M (2010). Meta-analysis of the diagnostic performance of stress perfusion cardiovascular magnetic resonance for detection of coronary artery disease. J Cardiovasc Magn Reson.

[10] Dietrich O, Reiser MF, Schoenberg SO (2008). Artifacts in 3-T MRI: physical background and reduction strategies. Eur J Radiol.

[11] Nayak KS, Cunningham CH, Santos JM, Pauly JM (2004). Real-time cardiac MRI at 3 tesla. Magn Reson Med.

[12] Cheng AS, Pegg TJ, Karamitsos TD, Searle N, Jerosch-Herold M, Choudhury RP (2007). Cardiovascular magnetic resonance perfusion imaging at 3-tesla for the detection of coronary artery disease: a comparison with 1.5-tesla. J Am Coll Cardiol.

[13] Gebker R, Jahnke C, Paetsch I, Kelle S, Schnackenburg B, Fleck E (2008). Diagnostic performance of myocardial perfusion MR at 3 T in patients with coronary artery disease. Radiology.

[14] Arnold JR, Karamitsos TD, Pegg TJ, Francis JM, Olszewski R, Searle N (2010). Adenosine stress myocardial contrast echocardiography for the detection of coronary artery disease: a comparison with coronary angiography and cardiac magnetic resonance. J Am Coll Cardiol Img.

[15] Meyer C, Strach K, Thomas D, Litt H, Nahle CP, Tiemann K (2008). High-resolution myocardial stress perfusion at 3 T in patients with suspected coronary artery disease. Eur Radiol.

[16] Klem I, Heitner JF, Shah DJ, Sketch MH, Behar V, Weinsaft J (2006). Improved detection of coronary artery disease by stress perfusion cardiovascular magnetic resonance with the use of delayed enhancement infarction imaging. J Am Coll Cardiol.

[17] Paetsch I, Jahnke C, Wahl A, Gebker R, Neuss M, Fleck E (2004). Comparison of dobutamine stress magnetic resonance, adenosine stress magnetic resonance, and adenosine stress magnetic resonance perfusion. Circulation.

[18] Klein C, Gebker R, Kokocinski T, Dreysse S, Schnackenburg B, Fleck E (2008). Combined magnetic resonance coronary artery imaging, myocardial perfusion and late gadolinium enhancement in patients with suspected coronary artery disease. J Cardiovasc Magn Reson.

[19] Ikuye K, Buckert D, Schaaf L, Walcher T, Rottbauer W, Bernhardt P (2013). Inter-observer agreement and diagnostic accuracy of myocardial perfusion reserve quantification by cardiovascular magnetic resonance at 3 Tesla in comparison to quantitative coronary angiography. J Cardiovasc Magn Reson.

